# Validation of the Paykel Suicide Scale and the Plutchik Suicide Risk Scale in Spanish Women during the Perinatal Period

**DOI:** 10.1155/2024/3741489

**Published:** 2024-07-23

**Authors:** Juan Miguel Martínez-Galiano, Sergio Martínez-Vázquez, Rocío Adriana Peinado-Molina, Antonio Hernández-Martínez

**Affiliations:** ^1^Department of Nursing of the University of Jaen, Jaen, Spain; ^2^Consortium for Biomedical Research in Epidemiology and Public Health (CIBERESP), Madrid, Spain; ^3^Department of Nursing, Physiotherapy and Occupational Therapy, Ciudad Real Faculty of Nursing, University of Castilla-La Mancha, Ciudad Real, Spain

## Abstract

**Introduction:**

No specific instruments are available to detect the risk of suicide in women during the perinatal period. Suicide in perinatal women is little studied despite being one of the main causes of maternal mortality. Its prevalence has increased by 100% in the United States in a decade. Moreover, it has consequences for the mother and the newborn.

**Objective:**

To validate the Paykel Suicide Scale and the Plutchik Suicide Risk Scale in Spanish women during the perinatal period.

**Materials and Methods:**

Cross-sectional study with Spanish women who were pregnant or who had given birth less than 18 months ago. Information was collected on sociodemographic variables, obstetric variables, and the newborn. A questionnaire that included both suicide scales and the Edinburgh Postnatal Depression Scale (EDPS) was used. An exploratory factor analysis (EFA), convergent and criterion validation, as well as internal consistency analysis, were performed.

**Results:**

In total, 237 women participated. The EFA determined that in the Plutchik Suicide Risk Scale 4 components explained 54.8% of the variance, while in the Paykel Scale, a single component explained 53.0% of the variance. In the convergent validity, the risk of suicide or suicidal ideation was related to the Plutchik Suicide Risk Scale and the Paykel Suicide Scale, respectively, with the variables: perception of low social support, risk of intimate partner violence, level of anxiety, history of mental pathology, and having experienced a stressful event in the last year, among others (*p*  < 0.05). The area under the receiver operating characteristic curve for the Plutchik Suicide Risk Scale scores was 0.88 (95% CI: 0.82–0.93), and for the Paykel Scale, it was 0.90 (95% CI: 0.85–0.95). The value of Cronbach's alpha (*α*) was placed for the Plutchik Suicide Risk Scale at 0.806 and for the Paykel Suicide Scale at 0.766.

**Conclusion:**

Both scales presented adequate psychometric characteristics to be used as a screening instrument for suicide risk in Spanish women in the perinatal period.

## 1. Introduction

Suicide is one of the leading causes of death worldwide and is considered a major public health problem [1, 2]. The prevalence of this phenomenon is also relevant during the perinatal period [3, 4]. In a recent systematic review with data from eight European countries (Denmark, Slovakia, Finland, France, Italy, the United Kingdom, the Netherlands, and Norway), suicide, together with cardiovascular diseases, was presented as one of the leading causes of maternal mortality [3]. In developed countries, suicide in the perinatal period represents between 5% and 44% of maternal deaths, and only 50% of women are identified [4].

Suicidal ideation is considered one of the main predictors of suicide [5, 6]. The prevalence of suicidal ideation within the perinatal period varies considerably depending on the type of study, population, measurement instrument, and perinatal stage, standing in a recent meta-analysis of 71 studies at around 8% [7]. Other studies found figures between 7% and 12% [4]. In the United States, rates of suicidal ideation during pregnancy increased 100% in the decade between 2008 and 2018 [8].

Suicidal ideation is not only a predictor of suicide, but it has also been associated with worse maternal and neonatal outcomes such as prematurity, low birth weight, congenital anomalies, respiratory distress syndrome, up to a sixfold increase in early fetal losses, and predicts effects adverse effects on child development and the mother–child bond [4, 5].

Currently, there is no specific validated instrument that allows screening for the risk of suicide in women during the perinatal period [4, 9, 10, 11, 12, 13, 14], so that it can be included and carried out by the professionals in charge of the regular follow-up and care during pregnancy, childbirth, and the postpartum period. In developed countries, a high percentage of maternal deaths are due to suicide [4], becoming one of the leading causes of maternal mortality [3, 4]. Despite its frequency, only half of women are detected [4]. The literature and clinical practice guidelines for the prevention and treatment of suicidal behavior recommend the use of specific instruments [4, 9, 10, 11, 12, 13, 14]. Among the main limitations observed in the few published studies is the use of different tools, providing nonspecific information about the study phenomenon and its severity in some cases. For example, Reid et al. [15] found that most studies used the Edinburgh Postnatal Depression Scale (EPDS) [16], where item 10, which says, “I have thought about harming myself” in the past 7 days, which can be interpreted in one of three ways: self-harm with suicide attempt (i.e., suicidal ideation), intentional self-harm without suicide attempt, unintentional self-harm (e.g., accidentally falling down stairs). Something similar occurs with the Patient Health Questionnaire-9 (PHQ-9) [17], where item 9 is used, both scales only includes an item that asks about suicidal ideation to evaluate the trend to suicidality. Authors using these tools also often do not provide a measure of the severity of suicidal ideation/self-harm. There are two specific tools that provide professionals with the capacity to detect the risk of suicidal behavior and previous suicide attempts, the intensity of current suicidal ideation, and other aspects related to suicide attempts: the Paykel Suicide Scale [18] and the Plutchik Suicide Risk Scale [19]. These scales do not require much time to complete and are also self-administered, easy to apply and interpret by health professionals. Considering all the above, we aimed to validate the Paykel Suicide Scale and the Plutchik Suicide Risk Scale in Spanish women during the perinatal period.

## 2. Materials and Methods

### 2.1. Design and Subject Selection

A cross-sectional study was carried out with women in the health coverage area of Jaén during the first quarter of 2023. Inclusion criteria were as follows: being pregnant with a gestational age ≥6 weeks or having given birth less than 18 months ago. This is the period where the need for the instrument is identified in the literature, thus the perinatal period. Exclusion criteria included women who could not communicate in Spanish (i.e., a language barrier as the questionnaire was administered in Spanish) and women under 18 years of age.

The required sample size was estimated using the factor analysis criteria, which requires 10 subjects for each item [20]. As the Plutchik Suicide Risk Scale, the scale with the greatest number of items, contains 15 items, a sample size of at least 150 subjects was required.

The questionnaire was distributed by collaborating health personnel, including midwives, nurses, and doctors. In the hospital, these were distributed in pregnancy follow-up consultations, in maternal and postpartum wards, and in the delivery room. In medical centers, questionnaires were distributed by the midwives who monitor the pregnancy and the puerperium, as well as in the neonatal checkups.

After women were selected and signed consent to participate, they were given the instructions to fill out the questionnaire. Health professionals at the women's consult or service provided support where needed to be able to solve any questions or issues that arose when completing the questionnaire. Collaborating professionals also used a WhatsApp group to discuss any issues that arose and thus be able to harmonize responses to the same question in all the participating centers. The sample was nonprobabilistic.

### 2.2. Information Sources

Data were collected using a previously piloted questionnaire that was distributed to the different hospitals and medical centers. For information collection, a self-made questionnaire was used that contained open and closed questions, which was given to the women. The language used in the questionnaire was understandable for all educational levels. Information was collected on sociodemographic variables (age, income level, etc.), lifestyles (alcohol consumption, smoking, etc.), obstetric history, family and personal history, including physical and mental condition, information on pregnancy and childbirth (planned pregnancy, pathology physical and/or mental in pregnancy, type of birth, complications, etc.) and on the newborn (health status, etc.). In addition, Generalized Anxiety Disorder-7 (GAD-7) screener [21, 22], Duke Scale of social support [23, 24], EPDS [16, 25, 26], and the PHQ-9 [17, 27] were included.

To determine the risk of suicide, several experts were consulted so that, based on the criteria that it was an instrument applicable by health professionals who are not specialists in mental health and/or self-administered by the woman, easy to apply and interpret, they would recommend which instrument was the most appropriate. The presence of suicidal ideation was assessed using two instruments: the Paykel Suicide Scale [18] and the Plutchik Suicide Risk Scale [19]. The Paykel Suicide Scale aims to assess suicidal ideation. Specifically, it explores thoughts of death (items 1 and 2), suicidal ideation (items 3 and 4), and suicide attempts (item 5). Although, as its name indicates, this tool assesses suicide, the truth is that some of its items ask questions about suicidal behavior (e.g., item 5). It consists of five items with a dichotomous response system, Yes/No (score 1 and 0, respectively). Scores range from 0 to 5 points. The time frame to which the questions refer is the last year. The higher the scores, the higher the frequency and severity of suicidal ideation. [28] For this study, we have considered two cutoff points: a more sensitive one taking into account any positive answer in any of the five items and another more specific cutoff point where it is considered positive if there are affirmative answers in items 3, 4, and 5. The second tool to validate was the Plutchik Suicide Risk Scale, a self-administered instrument consisting of 15 items with dichotomous responses (Yes/No), where each affirmative response adds 1 point. The scale score ranges from 0 to 15 points. A higher risk is indicated by a higher score. The authors of the validation in Spanish identified a score equal to or greater than 6 points as the cutoff point. Both scales are validated and have been used in the general Spanish population and in specific groups such as adolescents [29, 30]. The internal consistency (IC) of the instrument was found to be satisfactory, with a Cronbach alpha (*α*) value of 0.84 [19].

### 2.3. Data Analysis

Qualitative variables were described using absolute and relative frequencies, and quantitative data were described using means and standard deviation (SD).

Next, construct validity, convergent validity, and criterion validity were studied. Regarding construct validity, we carried out an exploratory factor analysis (EFA) to establish the underlying factors through the principal component analysis. However, first we ensured this analysis was applicable by using the Kaiser–Meyer–Olkin (KMO) tests and Bartlett's tests of sphericity. For the EFA to be appropriate, the KMO must be >0.6 and approaching 1. In addition, the Bartlett sphericity needs to be <0.05 to reject the null hypothesis of sphericity and confirm the suitability of the factorial model to explain the data. Varimax rotation was used in the EFA, and the Kaiser criterion was used to establish the number of factors to be retained, which is one of the most widely used criteria [31].

Within the construct validity, convergent validity was also analyzed to establish the relationship between the Paykel Suicide Scale, the Plutchik Suicide Risk Scale, and other factors that are associated with suicide risk, such as having a history of mental health problems, parity, having unintended pregnancies, presenting little social support, etc.

Data were analyzed by bivariate analysis using either Pearson's chi-square test, Fisher's exact test, or Student's *t*-test for qualitative or quantitative variable data, respectively. Statistical significance was set at *p*  < 0.05. To perform confirmatory analysis and determine the model fit, the IBM SPSS Amos program was used. Using as absolute fit measures: chi-square and root mean squared error of approximation (RMSEA); Incremental fit measures: The Comparative Fit Index (CFI), the Tucker–Lewis Index (TLI), and the Normed Fit Index (NFI);were also employed. Adjustment parsimony measures: parsimony ratio (PRATIO), Parsimony Comparative Fit Index (PCFI), Parsimony Normalized Fit Index (PNFI), and the Akaike Information Criterion Index (AIC). In order to interpret these indices, the critical values recommended by the literature were considered. These propose acceptable values above 0.90 for the TLI, CFI, and PRATIO indices, values above 0.80 for PCFI and PNFI, and below 0.08 for the RMSEA, as well as the lowest possible value for the AIC [32, 33, 34]. Once the aforementioned indicators had been determined, modification indices were requested, and the model was respecified by relating errors in order to redetermine the fit indicators.

In the absence of a gold standard suicide risk test and to study the criterion and predictive validity, a variable based on the combination of four criteria was created: positive scores in items three, four, and/or five of the Plutchik Scale, 6 points on the Paykel Scale, as well as positive scores for item number 10 of the EPDS Scale and number 9 of the PHQ-9. For this, a study of sensitivity and specificity was carried out with an analysis of the area under the ROC (receiver operating characteristic) curve (AUC).

The reliability analysis was performed using Cronbach's *α* to assess IC. However, due to the dichotomous nature of the responses of these scales, the KR20 coefficient (Kuder–Richardson) was employed [35]. The IC is used to assess the degree of correlation between two items and how well they measure the same concept. Cronbach's *α* is widely used to assess a scale's reliability [33] and has values that range from 0 to 1. Its values are interpreted as follows: *α* > 0.9 excellent, *α* > 0.8 good, *α* > 0.7 acceptable, *α* > 0.6 uncertain, *α* > 0.5 poor, and *α* < 0.5 unacceptable [32].

### 2.4. Ethical Considerations

The study has been approved by the Jaén Provincial Research Ethics Committee (VAPAYPE-23-002). The women received an information sheet for the participant and signed the informed consent.

## 3. Results

### 3.1. Participant Characteristics

A total of 237 women with a mean age of 35.4 years (SD = 5.28) participated, of which 21.9% (52) were pregnant and 78.1 (185) were postpartum. In this sample, 44.7% (106) had their first pregnancy, 12.7% (30) had an unplanned pregnancy, 29.4% (46) had some type of disease, and 23.2% (55) had a family history of suicide. Regarding the mean score of the Plutchik Scale, it was 2.68 points (SD = 2.86), with 16.8% (40) of women who presented scores of ≥6 points. For the Paykel Scale, the mean score was 0.70 points (SD = 1.21), being positive for any of the five items in 32.9% (78) and positive for any of the items 3, 4, or 5 in 12.7% (30) of the cases. The rest of the descriptive information can be found in Table 1.

### 3.2. Validity

#### 3.2.1. Convergent Validity

Next, convergent validity was studied through bivariate analysis between various variables that could predispose to suicide risk and the tools subject to validation through three variables: Plutchik Suicide Risk (low risk < 6 points/high risk ≥ 6 points), Paykel Ideation Risk (high risk: 3, 4, or 5 positive items) and the Paykel Risk “Ideation and thoughts” (high risk: positive in any item). In this analysis, we found a statistically significant association (*p* ≤ 0.05) with the three variables in the presence of a fetal anomaly, with the couple's relationship, with the EPDS Scale, the positive item 10 of the EPDS, the positive item 9 of the PHQ- 9, and the GAD-7 anxiety scale. A statistically positive association was also found for Plutchik Risk and Paykel Suicide Risk ideation and thoughts with the variables: planned pregnancy when a stressful event had occurred in the last year, previous mental health care, and the Duke social support scale. A detailed analysis is given in Table 1.

In this sense, the correlation was studied using the Pearson correlation coefficient between the two Plutchik and Paykel Scales (quantitative) with the Duke Scale scores (Plutchik: *r* = −0.554, *p*  < 0.001; Paykel: *r* = −0.381, *p*  < 0.001), EPDS (Plutchik: *r* = 0.761, *p*  < 0.001; Paykel: *r* = 0.567, *p*  < 0.001) and GAD-7 (Plutchik: *r* = 0.568, *p*  < 0.001; Paykel: *r* = 0.346, *p*  < 0.001). Finding a statistically significant correlation in all cases.

#### 3.2.2. Factor Construct Validity

The KMO test gave a value of 0.838 and 0.764 for the Plutchik and Paykel Scales, respectively, while Bartlett's sphericity test was <0.01 for both tools, so we proceeded to carry out the EFA.

**Figure 1:**
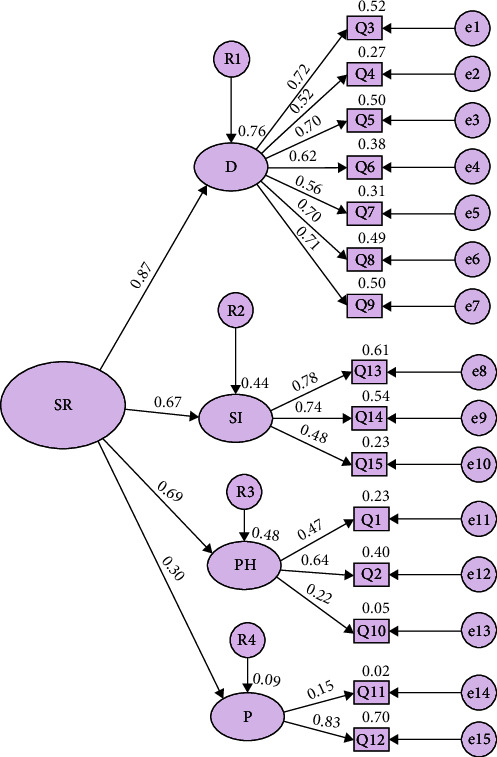
Path diagram scale Plutchik. SR, risk suicide; D, depression; SI, suicide ideation; PH, health problem-sleep; P, predisposition.

**Figure 2:**
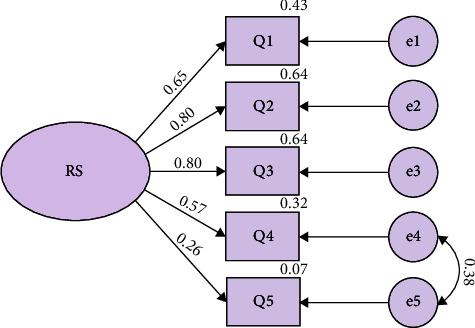
Path diagram Paykel Scale. RS, risk suicide.

Regarding the Plutchik Scale, four components explained 54.8% of the variance, while in the Paykel Scale, a single component explained 53.0% of the variance. The components of the Plutchik Scale were: component 1, “Depression,” consisted of items 3, 4, 5, 6, 7, 8, and 9; component 2, “Suicidal ideation,” consisted of items 13, 14, and 15; component 3, “Health problem-sleep” consisted of items 1, 2, and 10; while component 4, “Predisposition,” consisted of items 11 and 12. In the case of the Plutchik Scale, the diagonal anti-image correlations presented values between 0.515 and 0.911, while for the Paykel Scale, these were between 0.621 and 0.833.  Table 2 shows the scale items together with their respective factor weights. Figures 1 and 2 illustrate the path diagrams of the Plutchik Suicide Risk Scale and Paykel Suicide Scale.

#### 3.2.3. Internal Consistency

For the IC study, the *α* of the total of the questionnaire was used. Thus, for the Plutchik Scale had an *α* = 0.806 and the Paykel Scale an *α* = 0.766. On the Plutchik Scale, the greatest improvement in *α* was achieved by removing item 11, but only up to 0.828, while on the Paykel Scale, the greatest improvement in *α* was achieved by removing item 5, but only up to 0.789, so it was decided to keep them all. The values for each factor are shown in Table 3.

#### 3.2.4. The Confirmatory Factor Analysis

The confirmatory factor analysis revealed a poor fit of the single-factor models for both the Plutchik Scale and the Paykel Scale. However, the fit indices of the Plutchik model with four factors and the Paykel model, in which error 4 is related to error 5, exhibited a notable enhancement. This was evidenced by the global fit and incremental fit indices, although the parsimony indices remained unaltered.  Table 4 illustrates the values of each indicator and the criteria required to confirm the model fit.

#### 3.2.5. Criterion Validity

For criterion and predictive validity, we used a criterion variable (global suicide risk) made up of four variables: positive scores on items three, four, and/or five of the Paykel Scale, suicidal risk (≥6 points) on the Plutchik Scale, positive scores on item number 10 of the EPDS Scale, and positive scores on item number 9 of the PHQ-9 tool. The reason for using this criteria system is due to the variability in the degree of overlap of the various methods of determining suicide risk, as can be seen in Table 5, where there are only two cases where the women coincide in the four methods of determining suicide risk. This table shows that in 168 cases (70.9), none of the four methods used has a positive assessment.

**Figure 3:**
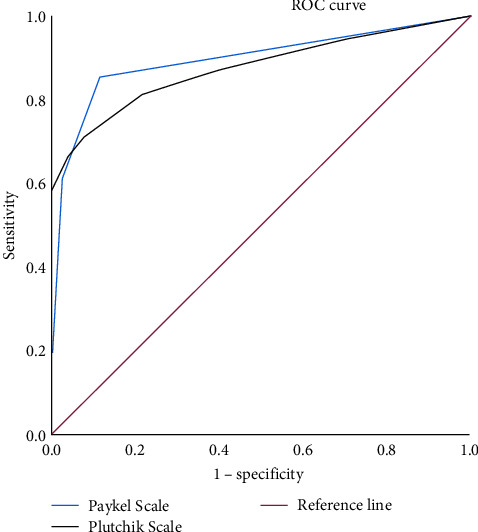
Area under the ROC curve of the Plutchik Suicide Risk and Paykel Scales to predict global risk of suicide.

Thus, we observed an area under the ROC curve for the Plutchik Scale scores of 0.88 (95% CI, 0.82–0.93) and for the Paykel Scale of 0.90 (95% CI, 0.85–0.95) to predict the overall risk of suicide with a good predictive capacity. The ROC curve can be seen in Figure 3.

## 4. Discussion

The psychometric characteristics of both the Paykel Suicide Scale and the Plutchik Suicide Risk Scale in the population of women who are in the perinatal period in Spain were adequate. The IC values, the convergent validity, the confirmatory factor analysis, as well as the criterion and predictive validity, showed that these scales are a reliable and valid tool to assess the risk of suicide in women during the perinatal period in Spain.

Regarding the possible limitations and bias, there may be a selection bias associated with nonresponse; however, there are no data or indications that suggest that the potential existence of this could have influenced the results. The number of women who declined participation was only 11. To our knowledge, there is no indication that these responses differ from those that did respond. The questionnaire was also anonymous, favoring an honest response and not a socially accepted or expected response; furthermore, women were permitted to decline to answer any questions they were uncomfortable with, especially when it comes to sensitive issues such as suicide or other aspects related to mental health, minimizing the possible biases associated with, although not entirely eliminated [36]. Women were given questionnaires to fill out at various healthcare settings, with support from health professionals as needed. The questionnaire was designed to be easily understood and distributed by midwives, nurses, and doctors. Any issues that arose were discussed in a WhatsApp group to ensure consistency in responses. We think this reduces the possible response bias. Among the strengths, it stands out that the sample size is notable, and it is also a representative sample of the reference population. The participation of women from different health centers in different geographical locations increases the probability of having an adequate representation of the population. It should be noted that this is a study that has validated suicide risk screening tools in women during the perinatal period, and no other study has been found in the literature.

The prevalence of suicide risk using the Plutchik Scale was 16.8%, and with the Paykel Scale, considering all the items, it was 32.9%, although if only the most restrictive criteria were applied considering “risk of suicidal ideation,” which only includes item 3, 4, or 5 of this scale, the prevalence was 12.7%. The prevalence figures are above those found in recent studies. [4, 7, 37] However, in the literature review carried out by Legazpi et al. [38], they identified prevalences of up to 18%, a figure higher than that of our study when the Plutchik Scale and the Paykel Scale were applied in their restrictive mode. It must be taken into account that the studies with which we compared ours applied variable and nonspecific instruments for the perinatal period and/or for the risk of suicide [15] and that this may have influenced these prevalence figures, this may be the reason for the difference with the one found by us [4, 7, 37, 38].

On the other hand, both scales presented an adequate convergent validity; thus, it coincides with previous studies that associate suicide risk and suicidal ideation with variables such as perceived low social support by the woman [38, 39, 40], having experienced a stressful life event in the last year [41], having a history of mental illness [41, 42], or experiencing intimate partner violence [37, 38, 43].

To assess criterion validity, a global suicide risk variable was created that included several variables used to identify women at risk of suicide or with suicidal ideation. These variables were the Paykel Scale [18] in its restrictive version, the Plutchik Scale [19], item number 10 of the Edinburgh postpartum depression scale [25], and also includes positive scores in item number 9 of the PHQ-9 tool [38]; finding very good ROC AUC values. It should be noted that no specific instrument has been identified to detect suicide risk or risk of suicidal ideation in women in the perinatal period. After all, some have been used in women in the perinatal period [7, 37, 38] with the purpose of identifying the risk of suicide; for this reason, it was decided to use them to assess the validity of criteria.

Finally, the IC of both scales was evaluated, identifying values that give a very good level of reliability. In the validation of the Paykel Scale in Spanish adolescents, the ordinal alpha was 0.93 [29]. We should remember that the ordinal alpha, as found in reference literature, is higher than Cronbach's alpha, which is the parameter used in our study [44]. Cronbach's alpha in the validation of the Plutchik Scale in the general population was 0.89, furthermore, a sensitivity of 0.81 [30] was found in Spanish nursing professionals [45], values very close to those found in our study.

The two scales are instruments that are easy to apply and interpret by the professionals in charge of the routine follow-up of pregnancy, childbirth, and the puerperium. Their implementation during perinatal follow-up can help to detect early women at risk of suicide and avoid the consequences that this can entail for the mother and the child. Notwithstanding, the Paykel Scale may be more ideal when recommending its use in pregnancy follow-up due to the number of items, being less time-consuming on its application, and this may make it easier to overcome some barriers by professionals since it is more time-efficient. For all these reasons, the systematic application of this scale would be very useful and potentially groundbreaking for women's health, just as the use of the EPDS is recommended.

In conclusion, the validation of the Paykel Scale and the Plutchik Suicide Risk Scale in Spanish women in the perinatal period showed adequate psychometric characteristics, making both tools appropriate for clinical practice in the Spanish setting.

## Figures and Tables

**Table 1:** Characteristics of the sample included in a validation study and their relationship with risk scores on the Plutchik Suicide Risk and Paykel Risk Ideation Scales.

Variable	Total	Plutchik Suicide Risk		Paykel Risk Ideation (positive items 3, 4, and 5)				Paykel Risk Ideation and thoughts (positive in any item)		
	*N* (%)	Score < 6, *N* = 197	Score ≥ 6, *N* = 40	*p*-Value	No, *N* = 207	Yes, *N* = 30	*p*-Value	No, *N* = 159	Yes, *N* = 78	*p*-Value
Maternal age				**0.020**			**0.006**			0.187
Mean (SD)	35.4 (5.28)	35.1 (5.04)	37.2 (6.05)		35.1 (5.16)	37.9 (5.49)		35.1 (5.32)	36.1 (5.14)	
Family income level				0.749			0.073			0.334
<1,000 euros	16 (6.8)	13 (81.3)	3 (18.8)		13 (81.3)	3 (18.8)		10 (62.5)	6 (37.5)	
1,000–1,999 euros	65 (27.4)	52 (80.0)	13 (20.0)		62 (95.4)	3 (4.6)		47 (72.3)	18 (27.7)	
2,000–2,999 euros	74 (31.2)	61 (82.4)	13 (17.6)		60 (81.1)	14 (18.9)		44 (59.5)	30 (40.5)	
≥3,000 euros	82 (34.6)	71 (86.6)	11 (13.4)		72 (81.1)	10 (12.2)		58 (70.7)	24 (29.3)	
Belongs to a religion				0.551			0.104			0.724
No	110 (46.4)	94 (85.5)	16 (14.5)		93 (84.5)	17 (15.5)		71 (64.5)	39 (35.5)	
Yes, but not practicing	96 (40.5)	79 (82.3)	17 (17.7)		89 (92.7)	7 (7.3)		67 (69.8)	29 (30.2)	
Yes, and practicing	31 (13.1)	24 (77.4)	7 (22.6)		25 (80.6)	6 (19.4)		21 (67.7)	10 (32.3)	
Intended pregnancy				**0.040**			0.480			**0.033**
No	30 (12.7)	21 (70.0)	9 (30.0)		25 (83.3)	5 (16.7)		15 (50.0)	15 (50.0)	
Yes	207 (87.3)	176 (85.0)	31 (15.0)		182 (87.9)	25 (12.1)		144 (69.6)	63 (30.4)	
Current illness				0.063			0.561			0.317
No	191 (80.6)	163 (85.3)	28 (14.7)		168 (88.0)	23 (12.0)		131 (68.6)	60 (31.4)	
Yes	46 (29.4)	34 (73.9)	12 (26.1)		39 (84.8)	7 (15.2)		28 (60.9)	18 (39.1)	
Pregnancy illness				**0.005**			0.698			0.182
No	142 (59.9)	126 (88.7)	16 (11.3)		125 (88.0)	17 (12.0)		100 (70.4)	42 (29.6)	
Yes	95 (40.1)	71 (74.7)	24 (25.3)		82 (86.3)	13 (13.7)		59 (62.1)	36 (37.9)	
Current status				0.608			0.455			0.529
Pregnant	52 (21.9)	42 (80.8)	10 (19.2)		47 (90.4)	5 (9.6)		33 (63.5)	19 (36.5)	
Postpartum	185 (78.1)	155 (83.8)	30 (16.2)		160 (86.5)	25 (13.5)		126 (68.1)	59 (31.9)	
Family history of suicide				0.481			0.986			0.769
No	182 (76.8)	153 (84.1)	29 (15.9)		159 (87.4)	23 (12.6)		123 (67.6)	59 (32.4)	
Yes	55 (23.2)	44 (80.0)	11 (20.0)		48 (87.3)	7 (12.7)		36 (65.5)	19 (34.5)	
Stressful event during last year				**<0.001**			0.159			**<0.001**
No	131 (55.3)	122 (93.1)	9 (6.9)		118 (90.1)	13 (9.9)		101 (77.1)	30 (22.9)	
Yes	106 (44.7)	75 (70.8)	31 (29.2)		89 (84.0)	17 (16.0)		58 (54.7)	48 (45.3)	
Number of pregnancies				0.313			**0.012**			0.280
Primigravida	106 (44.7)	91 (85.8)	15 (14.2)		99 (93.4)	7 (6.6)		75 (70.8)	31 (29.2)	
Multigravida	131 (55.3)	106 (80.9)	25 (19.1)		108 (82.4)	23 (17.6)		84 (64.1)	47 (35.9)	
Previous mental health care				**<0.001**			0.980			**0.005**
No	119 (50.2)	111 (93.3)	8 (6.7)		104 (87.4)	15 (12.6)		90 (75.6)	29 (24.4)	
Yes	118 (49.8)	86 (72.9)	32 (27.1)		103 (87.3)	15 (12.7)		69 (58.5)	49 (41.5)	
Admission to a mental health unit				0310*⁣*^*∗*^			1,000*⁣*^*∗*^			0551*⁣*^*∗*^
No	235 (99.2)	196 (83.4)	39 (16.6)		205 (87.2)	30 (12.8)		158 (67.2)	77 (32.8)	
Yes	2 (0.8)	1 (50.0)	1 (50.0)		2 (100.0)	0 (0.0)		1 (50.0)	1 (50.0)	
Relationship with partner during perinatal period				**0.004**			**0.008**			**0.004**
A lot of tension	9 (3.9)	4 (44.4)	5 (55.6)		5 (55.6)	4 (44.4)		2 (22.2)	7 (77.8)	
Some tension	96 (41.2)	80 (83.3)	16 (16.7)		82 (85.4)	14 (14.6)		61 (63.5)	35 (36.5)	
No tension	128 (54.9)	111 (86.7)	17 (13.3)		116 (90.6)	12 (9.4)		94 (73.4)	34 (26.6)	
Fetal anomaly during pregnancy				**0.043**			**0.014**			**0.032**
No	195 (82.3)	166 (85.1)	29 (14.9)		171 (87.7)	24 (12.3)		135 (69.2)	60 (30.8)	
Yes	18 (7.6)	12 (66.7)	6 (33.3)		12 (66.7)	6 (33.3)		8 (44.4)	10 (55.6)	
Missing	24									
Duke Scale				**<0.001**			0.502			**<0.001**
Mean (SD)	41.2 (10.83)	43.2 (9.84)	31.0 (9.87)		41.3 (11.11)	40.1 (8.79)		43.5 (9.86)	36.3 (11.2)	
EPDS Scale				**<0.001**			**0.004**			**<0.001**
Mean (SD)	9.9 (6.00)	8.1 (4.44)	18.5(5.28)		9.5 (6.02)	12.8 (4.98)		7.9 (4.37)	13.9 (6.78)	
EPDS Item 10				**<0.001**			**0.003**			**<0.001**
Negative	201 (84.8)	187 (93.0)	14 (7.0)		181 (90.0)	20 (10.0)		154 (76.6)	47 (23.4)	
Positive	36 (15.2)	10 (27.8)	26 (72.2)		26 (72.2)	10 (27.8)		5 (13.9)	31 (86.1)	
PHQ-9 item 9				**<0.001**			**0.090**			**<0.001**
Negative	211 (89.0)	190 (90.0)	21 (10.0)		187 (88.6)	24 (11.4)		157 (74.4)	54 (25.6)	
Positive	26 (11.0)	7 (26.9)	19 (73.1)		20 (76.9)	6 (23.1)		2 (7.7)	78 (92.3)	
GAD-7 Scale				**0.020**			**0.014**			**<0.001**
Mean (SD)	5.7 (4.86)	4.7 (4.39)	10.4 (4.17)		5.4 (4.76)	7.7 (5.11)		4.4 (4.13)	8.2 (5.24)	

*⁣* ^*∗*^Fisher exact test. Bold numerical values are statistically significant values (*p* < 0.05).

**Table 2:** Rotated component matrix Plutchik Suicide Risk Scale and Paykel Scale.

Item	1	2	3	4
Plutchik Suicide Risk Scale	Depression	Suicidal ideation	Health problem-sleep	Predisposition
Q1	—	—	0.485	—
Q2	—	—	0.650	—
Q3	0.731	—	—	—
Q4	0.628	—	—	—
Q5	0.792	—	—	—
Q6	0.568	—	—	—
Q7	0.636	—	—	—
Q8	0.591	—	—	—
Q9	0.717	—	—	—
Q10	—	—	0.729	—
Q11	—	—	—	0.643
Q12	—	—	—	0.763
Q13	—	0.776	—	—
Q14	—	0.802	—	—
Q15	—	0.641	—	—
Paykel Suicide Scale				

Q1	0.732	—	—	—
Q2	0.815	—	—	—
Q3	0.812	—	—	—
Q4	0.745	—	—	—
Q5	0.488	—	—	—

**Table 3:** Internal consistency.

Plutchik Suicide Risk Scale	Cronbach's alpha (*α*) KR-20
Total	0.806
When removing the item:	
(1) Do you usually take a medicine like aspirin or sleeping pills?	0.805
(2) Do you have difficulty falling asleep?	0.800
(3) Do you sometimes feel like you could lose control of yourself?	0.779
(4) Do you feel little interest in interacting with people?	0.797
(5) Do you see your future more pessimistically than optimistically?	0.786
(6) Have you ever felt useless or worthless?	0.786
(7) Do you view your future without hope?	0.797
(8) Have you ever felt like such a failure that you only want to stay in bed and give up everything?	0.778
(9) Are you currently depressed?	0.783
(10) Are you separated, divorced, widowed? And do you have unusual difficulty falling asleep or staying asleep?	0.811
(11) Do you know if any of your family members have tried to commit suicide before?	0.828
(12) Have you ever felt so angry that you could have been able to kill someone?	0.808
(13) Have you ever thought of committing suicide?	0.790
(14) Have you told anyone that you wished to commit suicide?	0.795
(15) Have you tried to kill yourself before?	0.802

Paykel Suicide Scale	

Total	0.766
When removing the item:	
(1) Have you felt that life is not worth it?	0.719
(2) Have you wished you were dead? For example, go to sleep and wish to never wake up.	0.669
(3) Have you thought about ending your life even if you were not actually going to do so?	0.673
(4) Have you ever reached the point where you really considered ending your life or made plans on how you would do it?	0.730
(5) Have you ever tried to end your life?	0.789

**Table 4:** Confirmatory factor analysis.

Indicators	Reference criteria	Unifactor model Plutnick Scale unifactorial	4 Factor model Plutnick Scale	Original model Paykel Scale	Model after relating errors Paykel Scale
Absolute fit indexes					
*χ*^2^	>0.005	<0.001	<0.001	<0.001	**0.967**
Root mean squared error of approximation (RMSEA)	<0.08	0.084	**0.056**	0.153	**0.000**
Incremental adjustment indexes					
Tucker–Lewis Index (TLI)	>0.90	0.787	**0.905**	0.835	**1.026**
Comparative Fit Index (CFI)	>0.90	0.818	**0.922**	**0.917**	**1.000**
Normed Fit Index (NFI)	>0.90	0.741	0.838	**0.905**	**0.998**
Parsimony adjustment indexes					
Parsimony ratio (PRATIO)	>0.90	0.857	0.819	0.500	0.400
Parsimony Comparative Fit Index (PCFI)	>0.80	0.701	0.755	0.459	0.400
Parsimony Normalized Fit Index (PNFI)	>0.80	0.635	0.755	0.453	0.399
Akaike Information Criteria Index (AIC)	Lowest possible	299.41	217.98	52.65	22.57

Assessment of model fit. The bold text indicates that the specified criteria have been met in the context of confirmatory factor análisis.

**Table 5:** Percentage match between the Paykel and Plutchik Suicide Risk tools and positive item 10 on the EPDS Scale and positive item 9 on the PHQ-9 Scale.

Paykel Suicide Scale (positive items, 3, 4, and 5)	Item 9 PHQ-9 positive	Item10 EPDS positive	Plutchik Suicide Risk Scale score ≥6	% Match
x	x	X	x	2 (0.8)
x	x	X		3 (1.3)
x	x		x	0 (0.0)
x		X	x	4 (1.3)
	x	X	x	15 (6.3)
x	x			1 (0.4)
x		X		1 (0.4)
x			x	2 (0.8)
	x	X		2 (0.8)
	x		x	2 (0.8)
		X	x	5 (2.1)
x				17 (7.2)
	x			1 (0.4)
		X		4 (1.7)
			x	10 (4.2)
				168 (70.9)

## Data Availability

The datasets generated during and/or analyzed during the current study are not publicly available but are available from the corresponding author upon reasonable request.

## Supplementary Materials

Supplementary 1 Checklist 1: STROBE statement—checklist of items that should be included in reports of observational studies.

Supplementary 2 Appendix 1: Spanish version of Plutchik Suicide Risk Scale (risk of suicide, RS) Paykel Suicide Scale (Paykel Suicide Scale, PSS).
