# Supplementary material for: Validation of the Paykel Suicide Scale and the Plutchik Suicide Risk Scale in Spanish Women during the Perinatal Period
Source: Depress Anxiety. 2024 Jul 23;2024:3741489. doi: 10.1155/2024/3741489 (PMC11919244; doi:10.1155/2024/3741489)
Supplement: Supplementary 2 — Appendix 1: Spanish version of Plutchik Suicide Risk Scale (risk of suicide, RS) Paykel Suicide Scale (Paykel Suicide Scale, PSS). [file 3741489.f2.docx]

**Appendix A: Spanish Version of Plutchik Suicide Risk Scale (Risk of Suicide, RS) Paykel Suicide Scale (Paykel Suicide Scale, PSS)**

**Plutchik Suicide Risk Scale (RS)**

Instructions: The following questions deal with things that you have felt or done. Please, answer each question with yes or no.

|  | **No** | **Yes** |
| --- | --- | --- |
| (1) Do you usually take a medicine like aspirin or sleeping pills? |  |  |
| (2) Do you have difficulty falling asleep? |  |  |
| (3) Do you sometimes feel like you could lose control of yourself? |  |  |
| (4) Do you feel little interest in interacting with people? |  |  |
| (5) Do you see your future more pessimistically than optimistically? |  |  |
| (6) Have you ever felt useless or worthless? |  |  |
| (7) Do you view your future without hope? |  |  |
| (8) Have you ever felt like such a failure that you only want to stay in bed and give up everything? |  |  |
| (9) Are you currently depressed? |  |  |
| (10) Are you separated, divorced, widowed? And do you have unusual difficulty falling asleep or staying asleep? |  |  |
| (11) Do you know if any of your family members have tried to commit suicide before? |  |  |
| (12) Have you ever felt so angry that you could have been able to kill someone? |  |  |
| (13) Have you ever though in committing suicide? |  |  |
| (14) Have you have told anyone that you wished to committ suicide? |  |  |
| (15) Have you tried to kill yourself before? |  |  |
| This is a scale that evaluates suicide risk. It comprises 15 items with a dichotomous reply (1 = yes; 0 = no), with a final score made up of the sum of the responses (the cut-off point in the Spanish population is 6 or more points). The higher the score obtained with this instrument, the higher the risk of suicide. | | |

**Paykel Suicide Scale (PSS)**

Please, mark with an x the box you consider best matches how you have felt or experienced during the last year.

|  | **No** | **Yes** |
| --- | --- | --- |
| (1) Have you felt that life is not worth it? |  |  |
| (2) Have you wished you were dead? For example, go to sleep and wish to never wake up. |  |  |
| (3) Have you thought about ending your life even if you were not actually going to do so? |  |  |
| (4) Have you ever reached the point where you really considered ending your life or made plans on how you would do it? |  |  |
| (5) Have you ever tried to end your life? |  |  |
| Instructions: This evaluates the severity of suicidal thoughts, differentiating two dimensions: thoughts of death and suicidal ideation. The items are arranged hierarchically (from least to most serious suicidal thinking). Thus, the cut-off points would be the following: no suicidal ideation (zero points), thoughts of death (positive score on items one or two), and suicidal ideation (positive score on items three, four and/or five). The higher the score, the greater the presence of suicidal ideation. | | |
